# Supplementary material for: The meaning of home questionnaire revisited: Psychometric analyses among people with Parkinson’s disease reveals new dimensions
Source: PLoS One. 2020 Dec 7;15(12):e0242792. doi: 10.1371/journal.pone.0242792 (PMC7721182; doi:10.1371/journal.pone.0242792)
Supplement: Attachment — Submitted filename: PLOSOne_Review1_Home Questionnaire_06-04-20.doc [file pone.0242792.s001.doc]

02-June-2020

PONE-D-20-10176

TITLE: “The Meaning of Home Questionnaire revisited: Psychometric analyses among people with Parkinson´s Disease reveals new dimensions”

**Reviewer:**

Comments to the Author

The authors aimed to create a psychometrically sound instrument to study perceptions of home in people with Parkinson’s disease (PD). This approach in environmental gerontology is novel and instruments for the assessment of perceptions of home are lacking. The authors collected data from a sample of 245 PD patients about perceptions of home captured by the Meaning of Home Questionnaire (MOH). Subsequently, they did a psychometric analysis of internal consistency, item-scale correlations and exploratory factor analysis. The found a new three-factor solution of MOH and satisfactory internal consistency level for the whole questionnaire. Overall, a solid piece of psychometric work resulting in the creation of a new instrument (MOH) for the assessment of perceptions of home in PD but possibly also in other clinical populations. However, I found some possible amendments or omissions and would beg the authors to improve or correct them. Therefore, I recommend a major revision.

MAJOR COMMENTS

**Introduction**

1. Could you please be more specific regarding the question of “perceived housing” and its clinical utility in PD or other diseases? What is supposedly the clinical utility of measurement of “perceived housing”?
2. Could you also be more specific in the formulation of “knowledge gap” regarding “perceived housing” in general and in PD in particular?

**Materials and Methods**

3. P. 8, l. 6: “The MOH is interview administered… “ I am sorry, I do not understand if MOH is a self-report scale or if it is an interview done by an expert on scaling with the patient (not a self-report)?
4. Second, who is allowed (what kind of professional) do the interview?
5. Third, the part of the sentence “The MOH is interview administered… ” does not seem to be grammatically correct.
6. Regarding Statistical analyses and reliability (internal consistency) in particular, I would welcome also other internal consistency measures (beyond Cronbach’s alpha) in accordance with EFPA guidelines for psychological and educational tests (Version 4.2.6; <http://www.efpa.eu/professional-development/assessment>), suggested by the Board of Assessments of the European Federation of Psychologists’ Associations (EFPA, 2013).
7. Can you add also L-Dopa equivalent and other medication in your descriptive statistics of PD sample?

**Discussion**

In Discussion (also in the analysis of results), I am missing besides the interpretation of the psychometric analysis some discussion of relation (e.g., a simple correlation analysis) between clinical measures of PD (L-Dopa equivalent, H-Y staging, PD duration) and MOH.

8. Why was MOH tested in PD sample?
9. And is MOH related to some clinical aspects of the disease?
10. What is the difference between perception of home and ADL/IADL measures? These, in my opinion, may also include some aspects of perception of home. I mean, why is the perception of home an independent construct and should be measured independently from ADL and everyday functioning measures?

MINOR COMMENTS

None.

**Tables**: No comments.

**Figures**: A higher dpi would make the inspection easier, the figure is blurred.
