# Supplementary material for: The meaning of home questionnaire revisited: Psychometric analyses among people with Parkinson’s disease reveals new dimensions
Source: PLoS One. 2020 Dec 7;15(12):e0242792. doi: 10.1371/journal.pone.0242792 (PMC7721182; doi:10.1371/journal.pone.0242792)
Supplement: Attachment — Submitted filename: Response to Reviewers.docx [file pone.0242792.s003.docx]

2020-11-05

Dear Editor in Chief,

Please find enclosed the revised version of our manuscript with the title *“The Meaning of Home Questionnaire revisited: Psychometric analyses among people with Parkinson´s Disease reveals new dimensions" (PONE-D-20-10176).*

We thank the reviewer for the final comment for improvements of our paper. Below follow our point-by-point responses to the comments of the reviewer, and a description of the revisions in the manuscript. In the uploaded revised manuscript version, all changes made are highlighted in yellow.

Alongside the revision based on the reviewers´ suggestions, we made minor text optimization throughout (however, not marked in the revised version of the manuscript) and uploaded our figure in PACE as recommended.

We hope that the revisions and responses will be sufficient for our manuscript to be accepted for publication in PLOS ONE. We are looking forward to further communication on this matter.

On behalf of myself and the co-authors,

Nilla Andersson

First author, PhD student

**Comment from a reviewer:**

Comment 6. Clarify if the MOH is in public domain or if the instrument is copyrighted.

**Response:** Thank you for this suggestion; we have incorporated information on this in the manuscript under the method section, as follows:

*“Psychometric properties of the MOH questionnaire for use in the general population of older people have been reported earlier [6]. The questionnaire [32] is available in English, Swedish, and German versions for research application (on request only, to author F.O.).”*
